# Leveraging Digital Workflows to Transition the Orthotics and Prosthetics Profession Toward a Client-Centric and Values-Based Care Model

**DOI:** 10.33137/cpoj.v6i2.42221

**Published:** 2023-12-22

**Authors:** C.F Hovorka

**Affiliations:** 1 Center for the Intrepid, Department of Rehabilitation Medicine, Brooke Army Medical Center, San Antonio, TX, USA.; 2 Defense Health Agency, Falls Church, VA, USA.; 3 Oak Ridge Institute for Science and Education, Oak Ridge, TN, USA.

**Keywords:** Healthcare, Client-Centric, Values-Based, Digital Workflow, Digital Technology, Competency, Curriculum, Orthotics, Prosthetics

## Abstract

The orthotics and prosthetics (O&P) profession has a history of responding to market demands in a reactive rather than proactive manner. This has created significant impacts including shrinkage in scope of practice and constraint in remuneration for professional services due to a fee-for-device third party payer system. Rapid changes in technology and healthcare combined with an outdated device-centric reimbursement system are creating unprecedented challenges that threaten sustainability of the O&P profession. Hence, a reassessment of the value of O&P care, and the O&P workflow process is necessary to inform an update to the value proposition and practice model for sustainability. This article reviews key factors contributing to the current state of O&P, and potential solutions involving an update in practitioner competencies, and the care delivery model (from device-centric to client-centric and values-based). Updates could be achieved by leveraging the use of digital workflows that increase efficiencies and enhance the value of clinical outcomes. Eventually, these updates could enable the O&P profession to elevate the value proposition that aligns with its most important stakeholders: client-patients and third-party reimbursement agencies in a rapidly changing technology and healthcare landscape.

## INTRODUCTION

This commentary was adapted from a presentation that was awarded Best Paper for Advancing Education at the 19th World Congress of the International Society for Prosthetics and Orthotics in Guadalajara, Mexico. It was also presented at the British Columbia Institute of Technology's Center for Applied Research and Innovation Workshop regarding the use of 3-D printing and digital workflows in the O&P and Assistive Technology professions. It addressed the question: “*How do professional O&P clinician education programs keep pace with changing technology and make informed decisions on what to include in curriculum and when?* Part 1 examines critical changes in healthcare and technology. Part 2 summarizes key challenges to the O&P profession that hinder the value of care. Finally, Part 3 proposes a solution that leverages the use of digital technologies in the O&P workflow process as a strategy to update the care delivery model to a client-centric and values-based approach and updates the role of the clinician as an O&P expert and healthcare technology manager.

### PART 1: Changes in healthcare and technology impose challenges to the O&P profession.

The Orthotics and Prosthetics (O&P) profession can only estimate the future healthcare economic determinants and market impacts and strategically position itself for change and likely cost-cutting measures ahead. In part 1, key challenges to U.S. healthcare that impact O&P are examined. A major challenge to the U.S. healthcare system is the persistent rise in expenditures which has influenced how healthcare is provided. Between 1970 and 2020, inflation-adjusted healthcare expenditures in the U.S. have risen from $300 billion to over $4 trillion and by 2030 are projected to exceed $6.7 trillion.^1,2^ Among the many reasons for this, five are notable including: a.) the rise in an aging population, b.) increasing multimorbidity, c.) proliferation of subspecialities, d.) culture of defensive medicine, and e.) increases in price/cost for care.^3–5^

#### a.) Rise in an aging population

Recently, the share of the U.S. population age 65 and over has risen to 16% and is projected to reach 20% by 2030.^6^ This is significant because aged persons over 65 spend more on healthcare than any other age group, hence growth in the number of older Americans is expected to increase total healthcare costs over time.^7,8^ Concomitantly, Medicare spending is projected to double over the next 30 years relative to the size of the economy.^3^

#### b.) Increasing multimorbidity

Over the past two decades, the U.S. population has experienced earlier onset of multimorbidity (>2 chronic diseases or medical conditions) which is associated with adverse health outcomes.^5,9–12^ In particular, the increase in multimorbidity burden has seen significant increases in cardiovascular, metabolic, endocrine, orthopedic, and behavioral disorders. Over the past decade, the onset of multimorbidity in the U.S. has occurred at comparatively younger ages beginning in midlife (e.g., persons aged between 40–50 years) resulting in an increasingly complex combination of conditions similar to older adults.^11,12^ This contributes to substantial costs for patients and health care systems.

For rehabilitation professionals, the dramatic rise in prevalence of multimorbidity, particularly obesity,^13,14^ cardiovascular disease, diabetes, arthritis, and cancer ^15^ create challenges in the complexity of client-patient assessment and treatment due to the number of overlapping body systems influenced by these conditions.^16,17^ Adding to the complexity of care are emerging epidemics in opioid addiction and mental health disorders.^18^

#### c.) Proliferation of subspecialities

In 2010, nearly 65% of physicians practiced in non-primary care subspecialties and the number of subspecialists is projected to grow an additional 21% by 2025.^19^ This is due in part to the increasing number and complexities of client-patients with multimorbidity. The proliferation of non-primary care subspecialists has produced a gap in primary care providers which is progressively being filled by nurse practitioner and physician assistant and expansion of their scope of practice.^19,20^ Substantial growth in non-physician providers is projected to increase to 43% of all non-primary care subspecialists by 2025.^19^ The impacts of these trends on O&P may influence the accuracy in referrals for O&P services as formal training and education in O&P for physicians, nurse practitioners and physician assistants is minimal.^21–23^

#### d.) Culture of defensive medicine

The practice of defensive medicine – a strategy of healthcare provision to adhere to standards of care in addition to reducing risk of litigation has contributed to redundancies in care delivery. This has been associated with a proliferation of diagnostic tests and other redundancies and inefficiencies in the care delivery process^24^ and may be associated with the decline of primary care physicians.^4,5^ These redundancies and inefficiencies contribute to increases in the costs for healthcare.

#### e.) Increases in price/cost for care

Increases in the price/cost for healthcare are due to a multitude of factors including those previously discussed that create a culture of fragmentation of care due to the multitude of subspecialist care providers delivering overlapping services with limited collaboration and communication.^1,16,17,25–27^ Given the persistent rise in healthcare expenditures and costs, is not surprising that alternative strategies and care delivery models are emerging as cost containment strategies,^27^ These strategies will be covered in Part 2.

### PART 2: U.S. Healthcare is transitioning toward client-centric and values-based care.

Client-centric and values-based care is part of a larger U.S. national quality strategy to reform how healthcare is delivered and reimbursed.^28^ The end goal of this approach is that third party payers incentivize and reimburse health care professionals for quality rather than quantity of services they provide.^29^ In O&P, the traditional device-centric model of care may minimize the wider value proposition for client centric and values-based care. The fee-for-device model of care in O&P not only constrains the economic sustainability of O&P provision of care, but it also erodes the value proposition by neglecting the impact on the client-patient's function and wellbeing. This is a major challenge for the O&P profession because it hinders the O&P profession in providing what reimbursement agencies want to know regarding the value of O&P service to the client-patient that answers key questions such as:

- How effectively does O&P service provision meet the client-patient's values, needs and goals?
- How effectively does O&P service provision improve the client-patient's health and reduce their disability including the costs for healthcare?

#### U.S. O&P Masters Curriculum Guidelines are device (not client) centric

Academic programs training O&P clinicians can influence the quality and capabilities of the workforce.^30^ Hence, the nature of the curriculum and training influences the capabilities of graduates and their ability to adapt to market demands. But when curriculum does not address market demands, challenges to its workforce become evident. This is the case with current U.S. O&P master's curriculum consisting of device-centric accreditation guidelines and standards for teaching O&P device design, manufacturing and fitting.^31^ Content on client-centric and values-based care principles and care provision methods is limited. Notable omissions in core curriculum are: **a.)** evaluating client-patient's additional body systems function beyond the muscular, neurological, skeletal and integumentary systems, **b.)** creating and prioritizing the client-patient problem list, **c.)** creating/defining client-patient goals and values using systematic and measurable strategies, **d.)** formulating and executing evidence-based treatment plans to achieve client-patient goals and values, **e.)** systematically assessing treatment outcome, and **f.)** updating the treatment plan as changes occur over the course of the client-patient's diseases or conditions, and **g.)** engagement in substantive scholarly activity. Further, there are limited guidelines regarding practitioner competencies (knowledge, skills, behaviors, traits) in the following key roles including: **a.)** research and scholarship, leadership, collaboration, communication, professionalism; and management of healthcare technologies.

Recently, the National Commission on O&P Education (NCOPE) spearheaded a summit meeting to evaluate the state of U.S. O&P curriculum.^32^ Stakeholders included were educators, accreditation agencies, clinicians and representatives from the scientific, business and credentialing agencies in O&P (e.g., NCOPE, American Board for Certification in O&P and Pedorthics, American Academy of O&P, American O&P Association). Unfortunately, other key stakeholders such as third-party payers, other healthcare and related professions (e.g., Medicine, Dentistry, Podiatry, Occupational Therapy, Physician Assistant, Nursing, Engineering) and O&P technology disruptors were omitted.

The goal of the meeting was to identify critical challenges to the O&P workforce and the requisite updates to curriculum needed to align the workforce for changes in technology and healthcare. Preliminary findings were that the device-centric model of reimbursement hindered the practice model of care and the need to consider methods that increase efficiencies in the O&P workflow.^32^

### PART 3: Leverage digital technologies that enable an update in the O&P curriculum to client-centric and values-based care.

Healthcare in the U.S. is moving from the traditional fee-for-service (e.g., fee-for-device in O&P) and volume-based reimbursement to one that is value based, due to national mandates.^33^ In order to improve value in health care delivery, we must improve the education for those providing health care.^34^ In Orthotics and Prosthetics, there is opportunity to update education through strategic curriculum changes that focus on strengthening a practitioner's skillsets in digital health technologies (i.e., data science, artificial intelligence) to enable evidence-based clinical decision-making, combined with the mastery of digital shape capture and additive manufacturing processes to improve efficiencies in the O&P workflow. By doing so, future O&P clinicians could solidify their role.^30^

**Figure 1:**
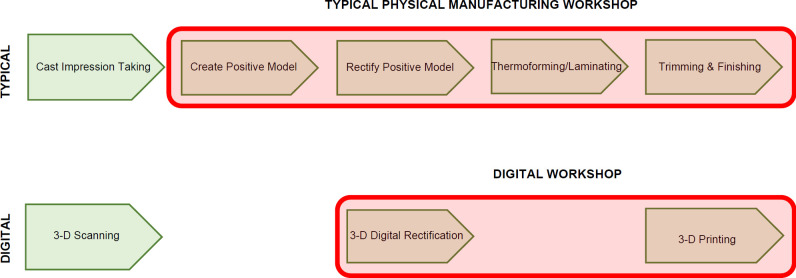
Comparison of workflow processes in Orthotics and Prosthetics. Typical artisanal workflow (top image) and digital workflow (bottom image).

Strategic implementation of digital health technologies (including 3-D shape capture, 3-D printing) has shown promise in improving efficiencies in the O&P manufacturing workflow compared to traditional artisanal workflow methods which require specific skills of the craft including substantial capital investment such as large overhead costs for production, machinery, equipment and skilled labor^35^ (**Figure 1**).

The digital workflow has the potential to enable efficiencies in time, resources and costs compared to the typical artisanal workflow process. Specifically, there is potential for digital workflow to improve efficiencies in the design and manufacturing process in O&P as these methods are innovative and highly scalable utilizing less subjective, more reproducible and potentially cost-effective methods.^36^ Advances in digital technology keep transforming healthcare, and from a training and education perspective these technologies have the advantage of providing more reliable feedback than traditional artisanal methods.^30,37–40^ Specifically, 3-D digital shape and rectification technologies can be used to characterize and quantify student competencies in executing task-specific methods in client-patient functional assessment and in the design and manufacturing process.^38^ Historical logs that quantify student performance in achieving milestones for shape capture, rectification and manufacturing quality can be quantified and characterized using digital records whereas traditional artisanal methods are less reliable and more subjective. Hence digital technologies can leverage new opportunities to target student learning and retention.^41^ As such, the potential for digital technologies to add utility and workflow efficiency creates opportunity to update other key elements of curriculum and education. This is discussed in the next section.

### FRAMEWORK 1: CanMEDS client-centered practitioner

Training and education of clinical care providers requires that students achieve a level of competence in knowledge, skills, abilities, and traits and demonstrating these safely and effectively within a scope of practice. Measuring competence is essential for determining the ability and readiness of healthcare professionals to provide quality services.^42^ Because the O&P Master's Curriculum Guide lists curricular topics and device-specific standards, a proposed competency-based framework that clearly identifies roles of the practitioner that enable service delivery could address gaps in content and conceptual framework of an O&P practitioner with transferrable knowledge, skills, abilities, and traits. A practitioner framework known as the Canadian Medical Education Directives for Specialists (CanMEDS) provides a practitioner competency framework developed by the Royal College of Physicians and Surgeons and contains many elements that can be adapted to the roles and competencies of an O&P practitioner.^43–45^ The CanMEDS is a set of competencies that are grouped thematically into seven roles (health advocate, communicator, collaborator, leader, scholar, professional and medical expert) deemed essential for effective healthcare provision. The framework is used for the education of physicians in Canada and is aimed at enhancing client-patient care and has been recognized globally. Each role is subdivided into key competencies that describe the knowledge, skills, abilities and traits in measurable and relevant elements that can be used to gauge student learning. Therefore, the CanMEDS practitioner framework provides a competency-based teaching structure that has the potential for more targeted and measurable learning outcomes.

**Figure 2:**
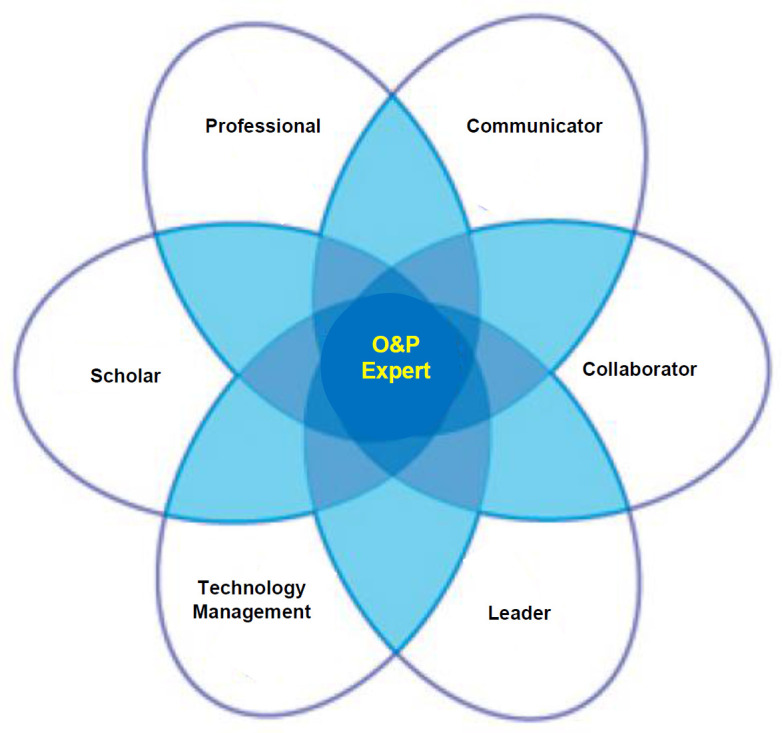
Modified CanMEDS practitioner framework for Orthotists and Prosthetists. The original practitioner framework created by the Royal College of Physicians and Surgeons was modified for O&P by updating the roles from “Medical Expert” and ‘Healthcare Advocate” to “O&P Expert” and “Technology Management”. Image adapted from the CanMEDS Physician Competency Diagram with permission of the Royal College of Physicians and Surgeons of Canada.

The CanMEDS framework can be adapted to the O&P clinical practitioner and scope of practice by replacing the roles of “Medical Expert” to “O&P Expert” and “Health Advocate” to “Technology Management” (**Figure 2**). These updates expand the O&P scope of practice for adapting to changes in technology and healthcare.

The Technology Management role expands the O&P practitioner competency for adapting to changes in technology.^45^ The O&P Expert is the integrator role that further expands the O&P practitioner competency required for managing the values, needs and priorities of the client-patient as well as other stakeholders such as the referral source and third-party payers. Hence, the role of O&P Expert requires the integration of all other roles (e.g., scholar, communicator, collaborator, professional, leader, technology management). As such, the revised O&P practitioner framework broadens the roles that includes medical-clinical focus and guidance of client-patients in navigating the increasing number of technology options.^44,45^

Two additional complimentary curricular frameworks (ICF and POP) are needed to address demands on the O&P clinician for the provision of client-centric and values-based care that includes the client-patient in decision making. One framework (ICF) is needed for clinician thoroughness by employing the International Classification of Functioning, Disability and Health (ICF) and another framework (POP) is needed for clinician efficiency by employing the Prosthetic Orthotic Process.

### FRAMEWORK 2: ICF client-centric and values-based care provision

**Figure 3:**
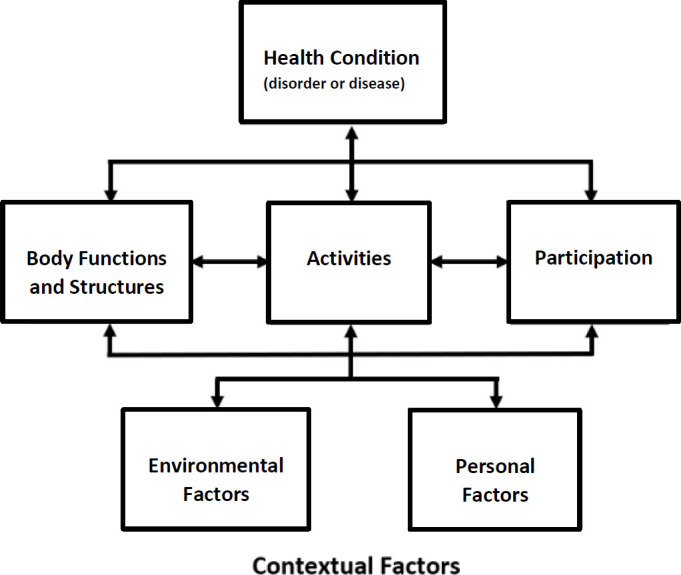
International Classification of Functioning, Disability and Health. Adopted from ICF, Geneva, Switzerland, World Health Organization, 2001; License CC BY-NC-SA 3.0 IGO

The International Classification of Functioning, Disability and Health (ICF)^46–48^ is a framework for understanding the “whole” client using a broader perspective. The IC views function and disability as an interaction between the person and his/her/their world (**Figure 3**). It is used as a clinical practice framework for O&P professionals to develop more informed client-centric and values-based perspectives. The approach has been implemented globally in O&P in Australia, Europe and India.^49–52^ It encourages the O&P practitioner to consider additional factors in the plan of care as part of a more comprehensive and holistic biopsychosocial perspective.

### FRAMEWORK 3: POP client-centric and values-based care provision

**Figure 4:**
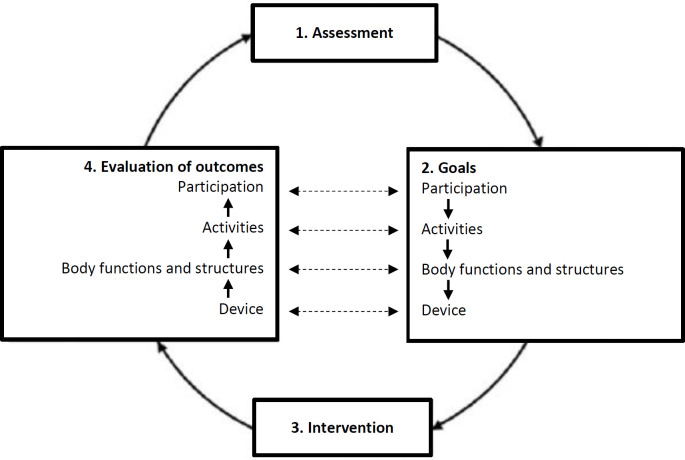
The Prosthetic and Orthotic Process. Adopted from Jarl G and Ramstrand N. A model to facilitate implementation of the International Classification of Functioning, Disability and Health into prosthetics and orthotics. Prosthet. Orthot. Int. 2018:42(5):248–275. DOI: 10.1177/0309364617729925

The Prosthetic and Orthotic Process (POP) is a service delivery framework that adapts the ICF to clinical O&P processes (**Figure 4**).^52^ Using this approach, the client-patient's goals related to activities are realized by an O&P treatment plan. This method utilized by the O&P professional, enables the client-patient to achieve goals related to body functions and structures. It consists of four steps in a cycle: **1.)** Assessment (including medical history and physical examination/review of systems of the client-patient. **2.)** Goals, specified on four levels including those related to participation, activity, body functions and structures and technical requirements of the O&P technologies. **3.)** Intervention, in which the appropriate course of action is determined based on the specified goal and evidence-based practice. **4.)** Evaluation of outcomes, where the outcomes are assessed and compared to the corresponding goals. After evaluation of goal fulfillment, a broad evaluation is then made including questions about the client-patient's satisfaction with the outcomes and the process. This evaluation determines if the process is ended or if another cycle in the process should be initiated.^52^

## CALL TO ACTION

Increasing costs of care and the need for cost cutting, the complexity of healthcare, and advances in technology, continue to challenge the O&P profession. There is an opportunity to proactively address gaps in the O&P master's curriculum that omit the wider scope of competencies to manage client-patients for client-centric and values-based care. These gaps could be addressed by including the modified CanMEDS competency-based framework to enhance the knowledge, skills, abilities and traits of the O&P practitioner. Two complimentary practice frameworks (ICF and POP) can further enable O&P practitioners to overcome increasing demands for thoroughness and efficiency required in modern healthcare by leveraging the use of digital technologies to improve efficiencies in the O&P workflow. Hence, the three frameworks can inform a more comprehensive approach for O&P client-centric and values-based care.

Transition of the proposed new curriculum has the potential to more effectively enable O&P clinicians to address the client-patient's values and priorities and select the optimal combination of technologies that meet client-patients' needs and to deliver the wider value proposition. Moreover, a modernized and reconceived practitioner as O&P Expert and Healthcare Technology Manager that directs greater attention to client-centric and values-based care would be able to distinguish themselves as a unique and valuable professional asset as an interdisciplinary health care team member. Therefore O&P education transition is crucial to the evolution of the profession, especially considering unprecedented challenges ahead.

## DECLARATION OF CONFLICTING INTERESTS

The views expressed herein are those of the author and do not reflect the official policy or position of the Brooke Army Medical Center, the Department of Defense, Defense Health Agency, or any agencies under the U.S. Government.

## SOURCES OF SUPPORT

No grants or support. The author was employed as a member of the faculty at Midwestern University, Glendale, AZ, USA during a portion of development of the three curricular frameworks.

## AUTHORS SCIENTIFIC BIOGRAPHY

**Figure:**
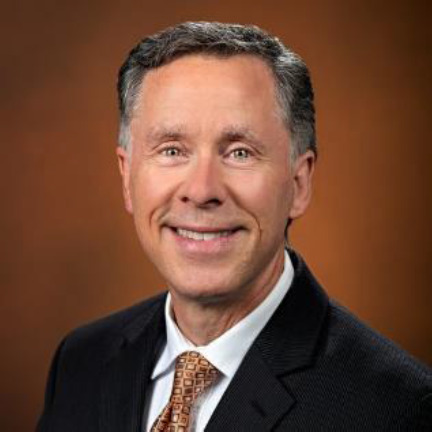


**Christopher F. Hovorka,** PhD, CPO, LPO, FAAOP. Dr. Hovorka completed Bachelor's degrees in Exercise Science (University of New Mexico) and Prosthetics and Orthotics (University of Washington), clinical residencies in Orthotics (Southern Illinois University School of Medicine) and Prosthetics (Connecticut Children's Medical Center, formerly Newington Children's Hospital), a Master's degree in Allied Health Science (University of Connecticut) and PhD in Applied Physiology with focus in Biomechanics and Neuromotor Control (Georgia Tech). He held faculty appointments at the University of Texas Southwestern Medical Center, St. Ambrose University, Georgia Tech, University of Pittsburgh, East Tennessee State University and Midwestern University. He has received continuous research grant funding for over 20 years, in areas ranging from cognitive/skills learning and curriculum development to the neuromechanics and clinical outcomes of persons using lower limb prostheses, orthoses and footwear. He also developed the nation's first accredited entry-level master's degree in Orthotics and Prosthetics (O&P) at Georgia Tech. Creation of that program sparked a national reassessment of the entry-level standards in O&P, and eventually adoption of the master's degree as the entry-level standard for O&P in the United States. Currently, he is an Established Scientist Fellow at the Center for the Intrepid, Department of Rehabilitation Medicine, Brooke Army Medical Center, San Antonio, TX, USA and is affiliated with the Defense Health Agency, Falls Church, VA, USA and Oak Ridge Institute for Science and Education, Oak Ridge, TN, USA.
